# Supplementary material for: Resolving the full spectrum of human genome variation using Linked-Reads
Source: Genome Res. 2019 Apr;29(4):635–45. doi: 10.1101/gr.234443.118 (PMC6442396; doi:10.1101/gr.234443.118)
Supplement: Supplemental Material [file supp_gr.234443.118_Supplemental_longranger-2.2.zip › longranger-2.2.tar.bz2!/lr2.2-public/lib/stan/cmdstan/stan/doc/api/html/functions_h.html]

Stan: Class Members


|  |  |
| --- | --- |
|  | Stan  2.5.0  probability, sampling & optimization |


- Main Page
- Related Pages
- Namespaces
- Classes
- Files

- Class List
- Class Index
- Class Hierarchy
- Class Members

- All
- Functions
- Variables
- Typedefs
- Enumerator
- Related Functions

- \_
- a
- b
- c
- d
- e
- f
- g
- h
- i
- j
- k
- l
- m
- n
- o
- p
- q
- r
- s
- t
- u
- v
- w
- x
- y
- z
- ~


All Classes Namespaces Files Functions Variables Typedefs Enumerations Enumerator Friends Macros Pages

Here is a list of all class members with links to the classes they belong to:

### - h -

- H()
  : stan::mcmc::base\_hamiltonian< M, P, BaseRNG >
- H0
  : stan::mcmc::nuts\_util
- hamiltonian\_
  : stan::mcmc::base\_hmc< M, P, H, I, BaseRNG >
- has\_denorm
  : std::numeric\_limits< stan::agrad::fvar< T > >
  , std::numeric\_limits< stan::agrad::var >
- has\_denorm\_loss
  : std::numeric\_limits< stan::agrad::fvar< T > >
  , std::numeric\_limits< stan::agrad::var >
- has\_flag()
  : stan::io::cmd\_line
- has\_high()
  : stan::gm::range
- has\_infinity
  : std::numeric\_limits< stan::agrad::fvar< T > >
  , std::numeric\_limits< stan::agrad::var >
- has\_key()
  : stan::io::cmd\_line
- has\_low()
  : stan::gm::range
- has\_quiet\_NaN
  : std::numeric\_limits< stan::agrad::fvar< T > >
  , std::numeric\_limits< stan::agrad::var >
- has\_signaling\_NaN
  : std::numeric\_limits< stan::agrad::fvar< T > >
  , std::numeric\_limits< stan::agrad::var >
- HasFloatingPoint
  : Eigen::NumTraits< stan::agrad::var >
- header
  : stan::io::stan\_csv
- help\_printed()
  : stan::gm::argument\_parser
- help\_width
  : stan::gm::argument
- HessianT
  : stan::optimization::BFGSMinimizer< FunctorType, QNUpdateType, Scalar, DimAtCompile >
  , stan::optimization::BFGSUpdate\_HInv< Scalar, DimAtCompile >
  , stan::optimization::LBFGSUpdate< Scalar, DimAtCompile >
- high\_
  : stan::gm::range
- highest()
  : Eigen::NumTraits< stan::agrad::var >


---

[ Stan Home Page ]

*© 2011–2014,
Stan Development Team.*
